# Efficacy of the Rotary Instrument XP-Endo Finisher in the Removal of Calcium Hydroxide Intracanal Medicament in Combination with Different Irrigation Techniques: A Microtomographic Study

**DOI:** 10.3390/ma13102222

**Published:** 2020-05-12

**Authors:** Jameela Denna, Lubna A Shafie, Loai Alsofi, Mey Al-Habib, Emad AlShwaimi

**Affiliations:** 1 Department of Endodontics, Faculty of Dentistry, King Abdulaziz University, Jeddah 21589, Saudi Arabia; dr.jameela-d@hotmail.com (J.D.); Maalhabib@kau.edu.sa (M.A.-H.); 2 Faculty of Oral and Dental Medicine, Cairo University affiliated to Faculty of Dentistry, King Abdulaziz University, Jeddah 21589, Saudi Arabia; dr.lubna.shafie@gmail.com; 3 Endodontic Division, Restorative Dental Sciences Department, College of Dentistry, Imam Abdulrahman Bin Faisal University, Dammam 31441, Saudi Arabia; ealshwaimi@iau.edu.sa

**Keywords:** Calcium hydroxide, micro-CT, passive ultrasonic irrigation, ProTaper Next, XP-endo Finisher

## Abstract

Objectives: This study aims to evaluate the efficacy of the rotary instrument XP-endo Finisher for the removal of Ca(OH)_2_ aided by different irrigation regimens. Methods: Sixteen double-rooted upper premolar human teeth were selected for the study. Thirty-two canals were prepared using a ProTaper Next rotary system up to X3. Then, the canals were filled with Ca(OH)_2_. The volume of Ca(OH)_2_ inside the canals was measured by microcomputed tomography (micro-CT). After that, the teeth were randomly allocated into two experimental groups, i.e., A and B (n = 16 canals). In group A, Ca(OH)_2_ was removed using the master apical file (X3). In group B, Ca(OH)_2_ was removed using a XP-endo finisher. In half of both groups (n = 8), syringe irrigation (SI) was used, while passive ultrasonic irrigation (PUI) was used for the other half. After removal, the remaining volume of Ca(OH)_2_ was measured. All data were statistically analyzed using two-way ANOVA with Tukey’s post hoc test. Results: The percentages of remaining Ca(OH)_2_ in the apical thirds of all canals were significantly higher as compared with the middle and coronal thirds in all groups (*p* < 0.05). There was no significant difference between different files and techniques (*p* > 0.05). Clinical Significance: This study presents a new method for the removal of Ca(OH)_2_ from root canals.

## 1. Introduction

Previous and recent endodontic technologies have focused on the reduction and eradication of microbes and microorganisms from the root canal system [1,2]. There is no available instrumentation method that can thoroughly disinfect the root canal system [2]. However, the placement of intracanal medication has been implemented to facilitate the disinfection procedure. Calcium hydroxide is extensively used as an intracanal medicament as it has antibacterial properties and its use is suggested for the treatment of infected canals or between root canal treatment visits.

Before root canal obturation, Ca(OH)_2_ medicament must be removed completely to allow adaptation of the obturation materials to the root canal walls [3]. Several studies have shown that it is difficult to completely remove Ca(OH)_2_ from root canals [4]. Remnants of Ca(OH)_2_ on the root canal walls can react with the endodontic sealer and change its properties [5]. Such changes include increasing its viscosity, reducing its flow, and affecting its setting time, and thus preventing sealer penetration and adhesion to dentinal tubules [1,6,7]. These changes affect the bond strength between the sealer and dentine [8]. A study by Kim and Kim, also found that residual Ca(OH)_2_ resulted in increased post-obturation apical leakage when a zinc oxide-eugenol root canal sealer was used [9]. The remnants could also react chemically with the sealer and affect the hermetic seal of the permanent root canal filling [10]. Furthermore, the solubility of Ca(OH)_2_ inside the root canal can cause voids on the dentine–filling interface that can enhance bacterial growth [11,12]. Therefore, the predictable and complete removal of Ca(OH)_2_ medicament before root canal obturation is crucial and is probably directly related to a successful treatment and favorable prognosis [13].

The removal of calcium hydroxide has been investigated using several products and protocols. The most commonly used protocol for the removal of Ca(OH)_2_ is the mechanical instrumentation using a master apical file (MAF) combined with sodium hypochlorite (NaOCl) irrigation [14]. In addition, several protocols have been recommended to improve the removal of Ca(OH)_2_, which include the use of NaOCl in combination with the chelating agent ethylenediaminetetraacetic acid (EDTA) and mechanical agitation provided by rotary files instrumentation or ultrasonic/sonic activation together with irrigation [14,15,16,17,18]. Margelos et al. reported that the best technique for calcium hydroxide removal from the root canal was flushing with NaOCl plus EDTA and filing, but even this method was unable to remove the material completely [7]. The use of rotary instruments or passive ultrasonic irrigation (PUI) has been found to remove more intracanal medicament as compared with conventional irrigation [14,19,20].

However, studies on different Ca(OH)_2_ removal protocols have shown that residual volumes ranging from 3% to 20% remained, mainly in the apical region [14]. Silva conducted a microtomographic study to assess the efficacy of passive ultrasonic irrigation (PUI) for the removal of calcium hydroxide medication with or without an additional file (F5). The results showed that the use of PUI was more efficient for the removal of Ca(OH)_2_ paste regardless of the use of the additional file. The highest residual volume in all techniques was in the apical region [20].

Wiseman showed that sonic and ultrasonic irrigation could not eliminate Ca(OH)_2_ from the mesial root canals of mandibular molars. Microcomputed tomography (micro-CT) scanning of the root canal system showed that the combination of rotary instrumentation and passive ultrasonic irrigation of 20 s for three periods significantly reduced the remnants of Ca(OH)_2_ as compared with sonic irrigation [21].

The XP-endo Finisher (FKG Dentaire, La Chaux de Fonds, Switzerland) is a rotary root canal instrument that has been introduced into the market. It is a universal NiTi-based instrument measuring ISO 25 in diameter with zero taper and it is indicated for use for instrumentation of canals with complex morphology and with inaccessible areas. The file is highly flexible and can expand. During use, the file reaches 100-fold of an equivalent sized file or 6 mm in diameter. These features help in dentine preservation. The new technology behind XP-endo Finisher files manufacturing is based on the shape-memory principles of the NiTi alloy. When the file is cooled, it becomes straight (M-phase). When the file is exposed to the body temperature inside the canal its shape changes to the A-phase, caused by its molecular memory. During rotation mode, the A-phase shape enables the file to access areas that are inaccessible with conventional instruments. According to the manufacturer’s guidelines, it can remove the medication inside the canal and the residual obturation material during retreatment.

The purpose of this study is to evaluate the efficacy of the finishing rotary instrument XP-endo Finisher in combination with different irrigation regimens for the removal of Ca(OH)_2_ from root canal dentin walls using computerized microtomography evaluation as compared with a master apical file (X3). 

## 2. Materials and Methods

### 2.1. Experimental Teeth Selection

Approval from the research ethics committee (090-10-17) was granted. Thirty-two double-rooted human teeth with completely formed apices were selected from a pool of teeth. The patient’s gender and age were unknown. Tooth selection criteria included teeth free of visible root caries, cracks, or fractures, and have completely formed root upon visual examination. The teeth were randomly coded and allocated blindly into two experimental groups. The external root surface was sealed with nail polish [22]. Buccolingual and mesiodistal periapical radiographs were taken to confirm the teeth had non-calcified canals.

### 2.2. Teeth Preparation

All teeth preparations were carried out at the Faculty of Dentistry, King Abdulaziz University, Jeddah, Saudi Arabia. Selected teeth were mounted in plastic tube holders with a radiolucent rubber base impression material. Access cavity preparation was done using round and tapered fissure carbide burs. The working length was determined by introducing a size 15 K-file in the canal up to the apical foramen, until the file was extruded from the apical foramen and 1 mm was subtracted from this length. Canal preparation was made using a ProTaper Next system (Dentsply Sirona, Ballaigues, Switzerland) (PTN, Dentsply Maillefer, Ballaigues, Switzerland) according to the manufacturer’s instructions after forming the glide path to full working length using a size 15 K-file. The files were powered by an electric motor (X-Smart plus, Dentsply Maillefer) with the manufacturer’s recommended rotational speed of 300 rpm and 200 g/cm torque. The MAF (X3) was used to form a minimal apical preparation with a dimension of 0.30 mm at the working length. After the preparation of each root canal, each file was carefully cleaned from debris.

## 3. Standard Irrigation Protocol

A standardized protocol was followed to irrigate all canals. The protocol included using two milliliters of 5.25% NaOCl to irrigate the canal after each instrument. After completing the instrumentation, 10 mL of 5.25% sodium hypochlorite NaOCl was used to irrigate the canals using a 30-gauge side-vented needle (Ultradent, South Jordan, UT, USA), with the cannula placed 2 mm short of the working length, followed by 3 mL of 17% EDTA (Sigma Lab Chem. Inc., Pitts- burgh, PA, USA) for one minute. A final rinse with 3 mL of 5.25% sodium hypochlorite (NaOCl) was performed, using a 30-G endodontic needle at 2 mm from the working length.

## 4. Placement of Ca(OH)_2_

All canals were dried using absorbent paper points (Dentsply Maillefer) and Ca(OH)_2_ (Ivoclar Vivadent) was inserted into the canals using a mechanically driven lentulo-spiral carrier (size 25, Dentsply Maillefer), adjusted to 3 mm from the WL. Radiographs were taken in two angulations, mesiodistal and buccolingual, to confirm that the canals were filled with Ca(OH)_2_. Access cavities were temporarily sealed with cotton pellets and cavit (3M ESPE Germany). Then, samples were saved in vials containing gauze saturated with saline at 37 °C for one week. Then, the specimens were scanned using microcomputed tomography scanning (micro-CT).

## 5. Removal of Ca(OH)_2_

### 5.1. Experimental Groups

**Figure 1:**
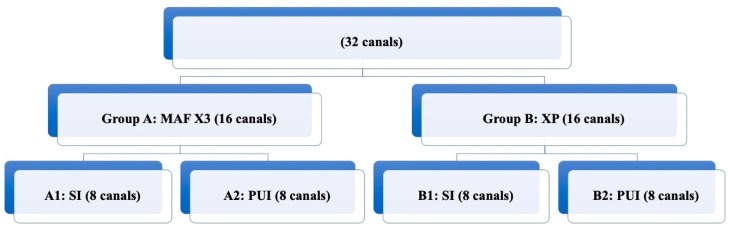
Distribution of different experimental groups according to the technique used for Ca(OH)_2_ removal.

After seven days, root canals were randomly distributed into two groups (*n* = 16), according to the procedure used for Ca(OH)_2_ removal (Figure 1).

#### 5.1.1. Group A: Ca(OH)_2_ Removal Using the Master Apical File MAF

The medicament was removed using the MAF (X3). This group was divided into two subgroups. In subgroup A1, the root canals were irrigated with 5.25% NaOCl (10 mL), and the MAF (X3) was inserted in rotary motion up to the WL. During its removal from the canal, the NaOCl solution was renewed. This procedure was repeated three times, and then the canal was filled with 17% EDTA (3 mL) for one minute which was replaced every 15 s, followed by a final rinse with 5.25% NaOCl (3 mL). In subgroup A2, after the use of the MAF (X3) file and regular irrigation, ultrasonic irrigation was performed for one min with an intracanal ultrasonic tip (Irri-Safe 20–25 mm thin intracanal, Satelec: Acteon group, Mérignac, France) 2 mm short from the WL. Ultrasonic activation was delivered for 20 s, twice during NaOCl irrigation and once during EDTA irrigation (mini Endo, SybronEndo, CA, USA). The total activation time was 60 s. The device was adjusted to 80% of maximum power. Then, the canals were dried with absorbent paper (35/4%; Dentsply Maillefer).

#### 5.1.2. Group B: Ca(OH)_2_ Removal Using the XP File

The medicament was removed using 5.25% NaOCl and the XP-endo Finisher. A contra-angle handpiece Element Motor (SybronEndo, Orange, CA, USA) was used. Root canals were each filled with 0.5 mL of 5.25% NaOCl. The instrument was adjusted to the WL, and then cooled down with Endo-Frost (Roeko, Langenau, Germany) according to the manufacturer’s instructions. After removal of the plastic tube, the instrument was inserted into the canal without rotation. Afterward, the instrumented was used in continuous rotation motion with 800 rpm and 1 N/cm torque. The instrument was used inside the canal for 1 min with slow 7–8 mm up-and-down movement the entire length of the canal. This procedure was repeated three times. Upon the instrument’s removal from the canal, the NaOCl solution was renewed. After removal of the instrument, a final irrigation protocol was performed using a 30-gauge side-vented needle with 10 mL of 5.25% NaOCl. Then, the canal was irrigated with 17% EDTA (3 mL) for one minute, followed by a final rinse with 5.25% NaOCl (3 mL). This group was divided into two subgroups. In subgroup B1, the XP file was used with regular irrigation technique. In subgroup B2, after the use of the XP file and regular irrigation, ultrasonic irrigation was performed using 5.25% NaOCl for one minute with an intracanal ultrasonic tip 2 mm short from the WL. The device was adjusted to 80% of maximum power. Then, the canals were dried with absorbent paper points (35/4%; Dentsply Maillefer).

### 5.2. Micro-CT Scanning Procedures and Evaluation Protocol

All micro-CT scanning and analysis were carried out at the College of Dentistry, Imam Abdulrahman Bin Faisal University, Dammam, Saudi Arabia. Two microtomographic scans were performed on each sample. The first scan was done one week after placement of the intracanal medicament and the second one was done after medicament removal. The teeth were mounted in a plastic tube holder with a radiolucent rubber base impression material. Scanning was performed using a micro-CT SkyScan 1172 machine (Belgium) with the following parameters: source voltage 90 v [23,24,25], source current (uA) = 112, image pixel size 13.73 [26,27,28], filter = Al + Cu, image format = tiff, exposure = 2900 [29], rotation step = 0.500 [30], frame averaging = 3, random movement = 10, and used 360° rotation. Then, raw images with tiff format were processed for reconstruction using NRecon Version 1.6.4.8, SkyScan 2011 software with the following settings: smoothing = 6, smoothing kernel = 2 (Gaussian), ring artifact correction = 6, beam hardening correction = 20%, and result file type = BMP. The CT scan version 1.11.10.0+ (64-bit) was used. SkyScan 2003-11 software was used for the calculation of the remaining calcium hydroxide in a cubic millimeter. The CT vol version 2.2.1.0 was used for realistic three-dimensional (3D) visualization.

### 5.3. Outcome Assessment

The mean volume of Ca(OH)_2_ before the removal was calculated. The higher greyscale value of Ca(OH)_2_ than that of dentine, allowed its identification by the manual segment procedure. The percentage of the volume of remaining Ca(OH)_2_ inside the canals after removal was calculated as (the mean volume of Ca (OH)_2_ before removal – the mean volume of Ca(OH)_2_ after removal) * 100/the mean volume of Ca(OH)_2_ before removal.

The calculation of Ca(OH)_2_ volume in each specimen was implemented using micro-CT software. Each dataset was also segmented using a uniform grayscale threshold to visualize and calculate the volume of residual Ca(OH)_2_ material. The volume of Ca(OH)^2^ is expressed as mm^3^. 

## 6. Statistical Analysis

The Shapiro–Wilk normality test was used to test the data distribution of Ca(OH)_2_ for the different groups. Two-way ANOVA with Tukey’s post hoc test was used for statistical analysis with statistical significance at (*p* < 0.05). The Prism 8 software (Version 8, GraphPad Software, La Jolla, CA, USA) was used for analysis. 

## 7. Results 

### 7.1. Descriptive Analysis

**Figure 2:**
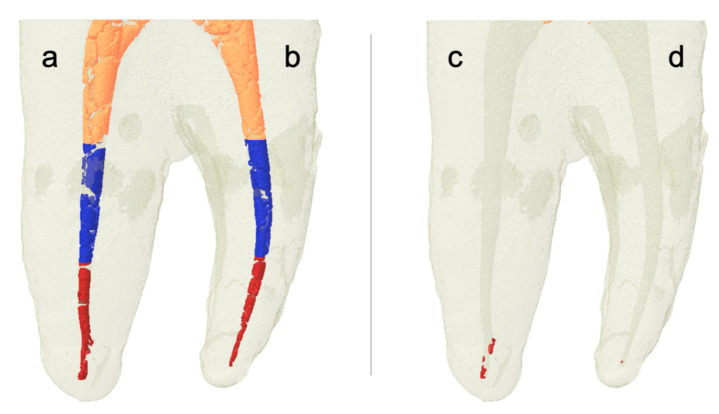
Three-dimensional rendered images constructed from microcomputed tomography (micro-CT) scans of root canals filled with Ca(OH)_2_. After placement (**a**,**b**); and after removal (**c**) master apical file and syringe irrigation (X3/SI); and (**d**) X3/passive ultrasonic irrigation (PUI). Note that the orange color indicates Ca(OH)_2_ in the coronal third, the blue color indicates Ca(OH)_2_ in the middle third, and the red color indicates Ca(OH)_2_ in the apical third.

**Figure 3:**
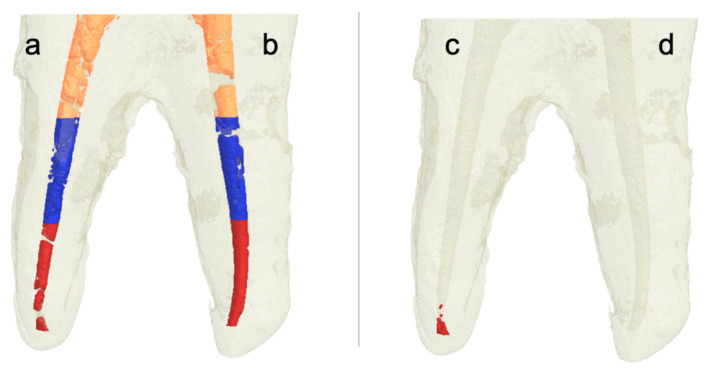
Three-dimensional rendered images constructed from micro-CT scans of root canals filled with Ca(OH)_2_. After placement (**a**,**b**); and after removal (**c**) XP/SI; and (**d**) XP/PUI). Note that the orange color indicates Ca(OH)_2_ in the coronal third, the blue color indicates Ca(OH)_2_ in the middle third, and the red color indicates Ca(OH)_2_ in the apical third.

**Figure 4:**
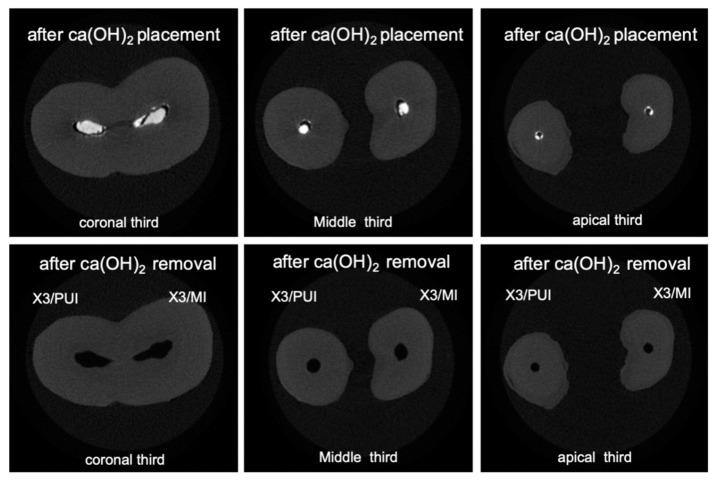
Cross-sectional micro-CT images of slices from coronal, middle, and apical thirds obtained before and after Ca(OH)_2_ removal. Top images represent cross-sections from different levels of the root canals after the placement of Ca(OH)_2_. Bottom images represent cross-sections from different levels of the root canals after the placement of Ca(OH)_2_.

**Figure 5:**
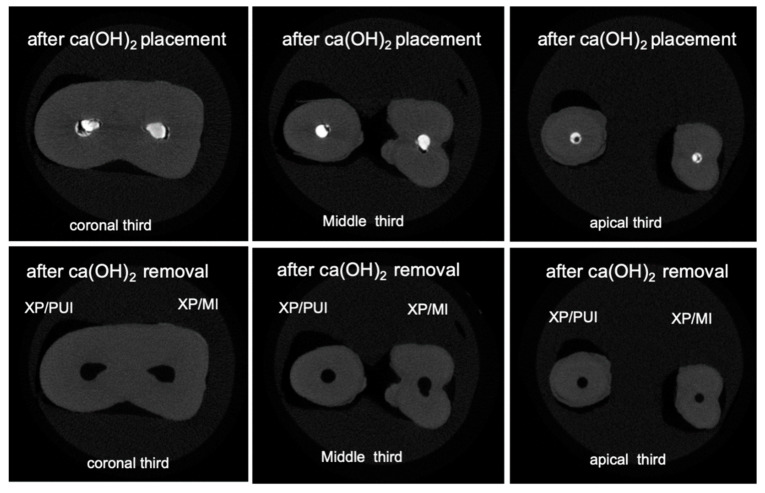
Cross-sectional micro-CT images of slices from coronal, middle, and apical thirds obtained before and after Ca(OH)_2_ removal. Top images represent cross-sections from different levels of the root canals after the placement of Ca(OH)_2_. Bottom images represent cross-sections from different levels of the root canals after the placement of Ca(OH)_2_.

Three-dimensional rendered images were constructed from micro-CT scans of root canals filled with Ca(OH)_2_, see Figure 2a,b and Figure 3a,b. Similar images were constructed from micro-CT scans of root canals after removal of Ca(OH)_2_, see Figure 2c,d and Figure 3c,d. We observe the complete removal of Ca(OH)_2_ from the coronal and middle thirds of root canals in all experimental groups. However, we notice remnants of Ca(OH)_2_ in root canals from different groups in the apical third, with almost complete removal for the group of canals in which the XP/PUI combination was used. Figure 4 and Figure 5 show cross-sectional images obtained from micro-CT scans of different levels (coronal, middle, and apical) from the different experimental groups before and after the removal of Ca(OH)_2_.

### 7.2. Quantitative Analysis

The efficacy of Ca(OH)_2_ removal from whole root canals after using the XP-endo Finisher and X3 files showed some differences when combined with either syringe irrigation (SI) or PUI. When X3/SI was used, the efficacy was 92.3% ± 11.2%, whereas when X3/PUI was used, the efficacy was 95.7% ± 4.5% (Table 1). This shows a slight increase in the efficacy when PUI was used, with no significant difference. When the XP-endo Finisher was used, the efficacy was calculated as follows: 94.3% ± 5.4% when XP/SI was used and 99.8% ± 0.002% when XP/PUI was used (Table 1). 

Calculation of the percentage of the remaining volume of Ca(OH)_2_ in different thirds of the root canals showed that there were significant differences in the apical third as compared with the middle and coronal thirds (*p* < 0.05). All techniques completely removed Ca(OH)_2_ from coronal and middle thirds of all root canals (Table 2). 

## 8. Discussion 

This study aimed to compare the efficacy of different protocols using X3, XP, PUI, and SI for the removal of Ca(OH)_2_ from the cleaned and shaped root canals. The widely used protocol for Ca(OH)_2_ removal is mechanical instrumentation using MAF combined with SI. Mechanical agitation provided by rotary files instrumentation or ultrasonic/sonic activation along with irrigation has been proven to be superior to SI in removing Ca(OH)_2_ from the root canal space. However, all the studies have shown that using these methods failed to completely remove Ca(OH)_2_ residues, especially from the apical third.

Complete removal of Ca(OH)_2_ from the middle and coronal thirds of root canals was observed in all experimental groups. However, complete removal of Ca(OH)_2_ from the apical third was not achieved in any of the experimental groups. The percentage of Ca(OH)_2_ residual volumes in the apical third ranged from 0.18% to 7.69%. This finding can be attributed to the normal anatomical morphology of the conical root canal system. The larger coronal diameter as compared with the middle and apical diameters, facilitates the irrigation and Ca(OH)_2_ removal from the coronal third [14,18]. Moreover, Ca(OH)_2_ tends to accumulate apically during the removal procedure, especially when apical anatomical variations are present [4]. In addition, the placement of the irrigation cannula and the ultrasonic tip 2 mm short of the working length, leaves this area without the direct effect of ultrasonic activation and limits the irrigation effect [13,21].

We noticed that the combined use of XP-endo Finisher with PUI resulted in almost complete removal of Ca(OH)_2_ from the root canals with the highest efficacy of 99.8% ± 0.002%. These results can be justified by the higher velocity and volume of irrigant flow created by passive ultrasonic irrigation, along with the increased flexibility of XP-endo Finisher and its ability to expand which makes it more efficient for the removal of Ca(OH)_2_ from root canals [31].

The results of our study are in agreement with those reported by Wiseman in that ultrasonic irrigation was more effective than sonic irrigation after instrumentation for removal of calcium hydroxide from the mesial root canals of mandibular molars with reported residual volumes ranging from 14% to 28% [21]. However, the reported residual volume sof Ca(OH)_2_ in our study were lower, ranging from 0.18% to 7.69%, which demonstrated a more efficient protocol for calcium hydroxide removal.

Another microtomographic study which was conducted to assess the efficacy of PUI for removal of calcium hydroxide medication with or without an additional file (F5) showed that the use of PUI was more effective for the removal of Ca(OH)_2_ paste regardless of the use of the additional file [20]. The reported residual volumes of Ca(OH)_2_ were in the same range of our study, ranging from 2.9% to 8.8%, with the highest residual volume in the apical region in all techniques similar to our findings.

Another study compared the effectiveness of five different instruments for the removal of Ca(OH)_2_ combined with irrigant agitation from simulated internal root resorption cavities, under scanning electron microscopy analysis; none of the instruments used was able to completely remove the Ca(OH)_2_ paste. However, the EDDY^®^ and XP-endo^®^ Finisher were more effective for the removal of Ca(OH)_2_ residues than the Ultrasonic, EndoActivator^®^, and XP-endo^®^ Shaper, which was in agreement with the results of our study [32]. 

In a similar study, in which only an optical microscopy was used for Ca(OH)_2_ residue analysis, the XP-endo Finisher file and PUI significantly removed more Ca(OH)_2_ than conventional needle irrigation, with no significant differences between them from artificial standardized grooves in the apical third of root canals [33]. 

Our results are also consistent with the results of Kfir et al. who showed that the XP-endo Finisher is a more superior method for the removal of Ca(OH)_2_ from the apical third [34]. The XP, which was intended to be used as a finisher file, also failed to completely remove the Ca(OH)_2_ from the canal. This could be due to the lack of enough contact time between the file and the canal wall during the one-minute window indicated by the manufacturer’s instructions. Further testing would be worthwhile by keeping the XP running longer or using it for multiple cycles to check if it could perform more effectively in Ca(OH)_2_ removal [33,34]. 

More research studies should be carried out to identify an irrigation protocol that could effectively remove Ca(OH)_2_ residues from root canal spaces.

## 9. Conclusions

For the removal of Ca(OH)_2_, there were no significant differences between the different method combinations. However, removal of Ca(OH)_2_ from the coronal and middle thirds was more efficient than from the apical third in all experimental groups. The XP-endo Finisher showed comparable efficacy to the Master Apical File for the removal of Ca(OH)_2_ regardless of the irrigation system used.

## Figures and Tables

**Table 1:** Efficacy of Ca(OH)_2_ removal using different techniques. The efficacy is presented as the percentage of the volume of removed Ca(OH)_2_ from the root canals after the use of different removal methods.

Technique Used	Efficacy%
X3/SI	92.3% ± 11.2%
X3/PUI	95.7% ± 4.5%
XP/SI	94.3% ± 5.4%
XP/PUI	99.8% ± 0.002%

**Table 2:** The mean percentage of the volume of remaining Ca(OH)_2_ in whole root canals and different thirds after using different removal techniques.

Technique Used	% of Remaining Ca(OH)_2_			
	Whole Canal	Coronal Third	Middle Third	Apical Third
X3/SI	7.69% ± 11.25%	0.02% ± 0.04%	0%	7.67% ± 11.26%
X3/PUI	4.24% ± 4.51%	0.008% ± 0.01%	0%	4.23% ± 4.51%
XP/SI	5.65% ± 5.37%	0%	0.02 ± 0.04%	5.63% ± 5.36%
XP/PUI	0.18% ± 0.27%	0%	0.05 ± 0.11%	0.12% ± 0.16%
